# Kikuchi-Fujimoto Disease: A Rare Condition Mimicking Sepsis

**DOI:** 10.22038/ijorl.2025.82006.3754

**Published:** 2025

**Authors:** Sethu Thakachy Subha, Naseeha Roslan Ainur, Mohd Ali Razana

**Affiliations:** 1 *Department of Otorhinolaryngology, Hospital Sultan Abdul Aziz Shah, Faculty of Medicine & Health Sciences University Putra Malaysia, Malaysia.*; 2 *Department of Pathology, Hospital Sultan Abdul Aziz Shah, Faculty of Medicine & Health Sciences University Putra Malaysia, Malaysia.*

**Keywords:** Fever, Kikuchi-Fujimoto disease, lymphadenopathy, Necrotizing Lymphadenitis, sepsis

## Abstract

**Introduction:**

Kikuchi-Fujimoto disease is a self-limited disease which is also known as histiocytic necrotizing lymphadenitis. The Kikuchi-Fujimoto disease is a rare cause of cervical lymphadenopathy, with or without systemic signs, such as fever, leukopenia, and skin rashes.

**Case Report:**

We presented a case of Kikuchi-Fujimoto disease in a 29-year-old female mimicking sepsis after a COVID-19 infection. Clinical examination revealed stable vitals and multiple diffuse non-tender bilateral cervical and axillary lymph adenopathy with hepatosplenomegaly. The patient’s blood parameters showed leukopenia, raised erythrocyte sedimentation rate, lactate dehydrogenase, microcytic hypochromic anemia with normal renal and liver function tests. The patient was treated with broad spectrum intravenous antibiotics and subcutaneous neupogen with a clinical suspicion of sepsis. The patient then underwent a CT scan which validated the clinical findings. Although the fever subsided, a persistent cervical lymphadenopathy was observed and the biopsy confirmed it to be necrotising lymph adenitis secondary to Kikuchi-Fujimoto disease. This patient has been regularly monitored and has shown resolution of cervical lymphadenopathy.

**Conclusion:**

Clinicians should suspect Kikuchi-Fujimoto disease when patients present with persistent cervical lymphadenopathy unresponsive to initial medical treatment. Lymph node biopsy should be undertaken to rule out Kikuchi-Fujimoto disease and prevent these patients from extensive diagnostic procedures and inappropriate treatment modalities.

## Introduction

Kikuchi-Fujimoto disease predominately occurs in young adult female patients (1). Various studies have shown that cervical lymphadenopathy with fever as the most common clinical manifestation of Kikuchi-Fujimoto disease (2,3). Other clinical symptoms include headache, vomiting, malaise, weight loss, arthralgia, and skin rashes (2). The aetiology of Kikuchi disease is unknown but infective and autoimmune conditions have been described as causative factors (2). A few case reports have also described Kikuchi disease as triggered or occurring along with COVID-19 and post-vaccination (4,5). Some previous studies have also reported a complex association between Kikuchi-Fujimoto Disease and systemic lupus erythematosus (6-8). Clinicians are not familiar with the Kikuchi-Fujimoto disease and this report highlights that a timely diagnosis of this self-limiting entity is crucial. 

## Case Report

**Fig 1:**
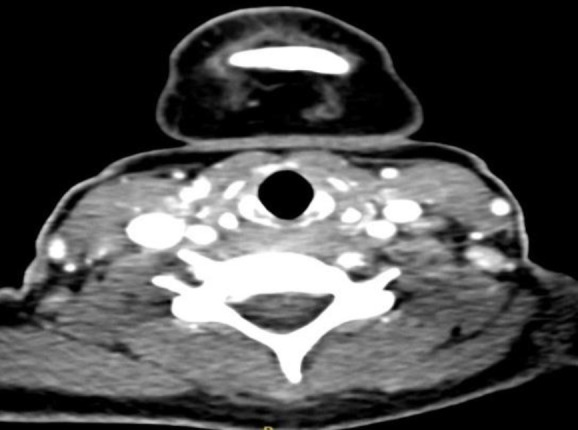
Contrast enhanced CT scan shows multiple lymph nodes with heterogenous enhancement on both sides of the neck with no necrotic component and some of them are matted.

**Fig 2:**
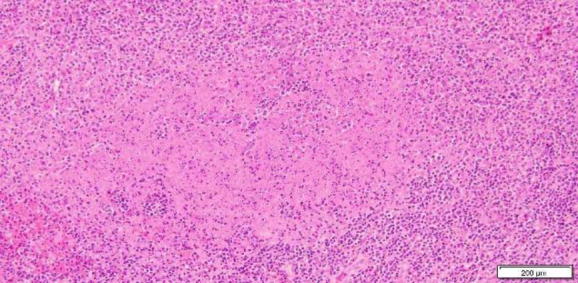
Photomicrograph of surgical specimen from lymph node shows amorphous necrotic debris and karyorrhectic nuclear dust (Hematoxylin-eosin stain, x 100 magnification).

**Fig 3:**
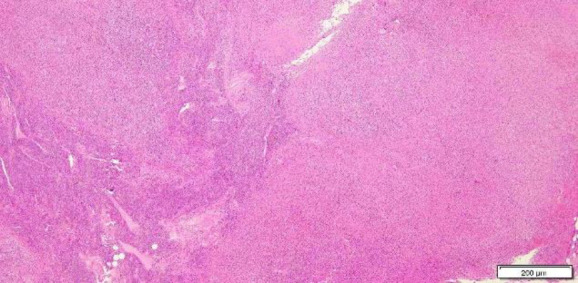
Photomicrograph of surgical specimen from lymph node shows partial involvement by irregularly shaped, pale areas containing eosinophilic granular material, and abundant karyorrhectic debris (nuclear dust) surrounded by mononuclear histiocyt

**Fig 4:**
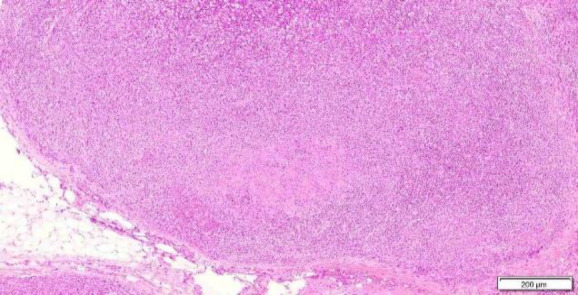
Photomicrograph of surgical specimen from lymph node shows pale areas and mottled or starry sky (upper part) appearance due to small lymphocytes admixed with immunoblasts. (Hematoxylin-eosin stain, x 40 magnification)

**Fig 5:**
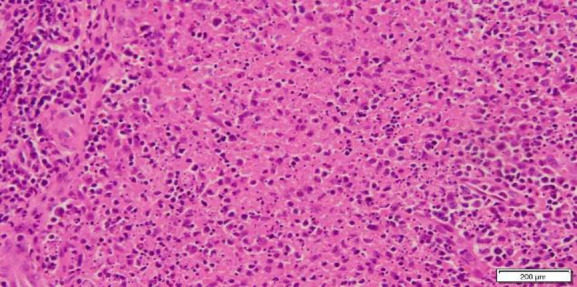
Photomicrograph of surgical specimen from lymph node shows central karyorrhectic debris with small lymphocytes, mononuclear histiocytes and plasmacytoid dendritic cells. No neutrophils. (Hematoxylin-eosin, x 200 magnification)

A 29-year-old female presented with a one-month intermittent fever, headache, and neck lumps. Her headache was nonspecific with no temporal pattern. The patient had a history of COVID-19 infection two weeks prior to the onset of the symptoms. A clinical examination showed stable vitals and multiple diffuse non-tender bilateral cervical and axillary lymph adenopathy and hepatosplenomegaly. The examination of the central nervous, cardiovascular, and respiratory systems was normal. Laboratory tests showed bicytopenia (white cell count-2.18 x 109/L), increased erythrocyte sedimentation rate, (65mm/hour), raised lactate dehydrogenase, and microcytic hypochromic anaemia with normal renal and liver function tests. The patient was initially treated for neutropenic sepsis with broad-spectrum antibiotics and subcutaneous neupogen. Blood and urine cultures remained negative. The patient’s fever had settled but the neck nodes persisted and were further investigated for lymphoma and autoimmune diseases. Assessments of antibodies against human immunodeficiency virus and hepatitis B and C, chest radiography, and electrocardiography were unremarkable. The antinuclear antibody test was positive. Further serological tests including rheumatoid factor, anti-double-stranded-DNA antibodies (dsDNA), and anti-extractable nuclear antigens (ENA) were performed, and all of them revealed negative results. A computed tomography of the brain, thorax, abdomen revealed diffuse cervical (Figure 1) and axillary lymphadenopathy with hepatosplenomegaly. Meanwhile, a biopsy of the neck node was performed and histopathology confirmed necrotising lymphadenitis secondary to Kikuchi’s disease, as shown in Figures 2, 3, 4, and 5. A written informed consent for publication was obtained from the patient. The patient remained well during the follow-up.

## Discussion

Kikuchi-Fujimoto disease (KFD) was first described in 1972 by Kikuchi and Fujimoto in Japan (2). A review of related literature review revealed that COVID-19 infection and vaccination have triggered the Kikuchi-Fujimoto disease and other autoimmune diseases, including systemic lupus erythematosus, rheumatoid arthritis, vasculitis, multisystem inflammatory syndrome in children and adults, and antiphospholipid syndrome (3,4,5,9). Although various viruses have been connected to KFD, none have been consistently associated with the disease. Similarly, COVID-19 has been linked to multiple inflammatory and immune-related conditions, but the underlying mechanism of this response is still limited. Research findings indicate that COVID-19 triggers the activation of immune cells and enhances the production of autoantibodies, contributing to the onset of autoimmune phenomena (3-5). The Kikuchi-Fujimoto disease most commonly affects young adults and it can occur in all racial groups (2). Due to its non-specific clinical features, KFD can mimic various clinical conditions (10). Similar to our case, the initial presentation may resemble sepsis but after a persistent lymphadenopathy, it can mimic tuberculosis, systemic lupus erythematosus (SLE), or lymphoma. Some published studies have highlighted the association between KFD and SLE, with 30% of the Kikuchi-Fujimoto disease cases occurred previously, while in 23% of the Kikuchi-Fujimoto diseases occurred after SLE and simultaneously as well (6-8). A study carried out by Imamura M et al. hypothesised that KFD, an unknown aetiology might reflect a self-limited, SLE-like autoimmune condition induced by virus-infected transformed lymphocytes. This might indicate a definite relationship and occurrence mechanism between KFD and SLE (7). Laboratory blood tests and imaging are nonspecific for KFD (11), while blood tests might show leucopenia, raised erythrocyte sedimentation rate, C-reactive protein levels, and lactate dehydrogenase. Autoimmune antibody tests like rheumatoid factor and antinuclear antibody studies are generally negative. On the contrary, the antinuclear antibody was positive in our patient. However, the patient never showed any signs of joint, oral, or cutaneous abnormalities, or any other serological tests for systemic lupus erythematosus (such as rheumatoid factor, antinuclear antibodies, and anti–double-strand DNA antibodies), which were consistently negative. Studies have shown that KFD can be rarely presented with positive antinuclear antibody, with a degree of clinical and morphological overlap between KFD and SLE. A positive antinuclear antibody can be found in a range of conditions and should always be interpreted in the context of clinical and other specific investigations. In the Kikuchi-Fujimoto disease, imaging findings are non-specific, with the majority demonstrating homogenous nodal enlargement and perinodal infiltration (2,12). A definitive diagnosis of Kikuchi-Fujimoto disease can be done through an excision biopsy of the lymph node and its histological findings (10). The characteristic cytomorphological features of KFD include karyorrhexis, small phagocytic histiocytes with sharply angulated nuclei, an increased number of immunoblasts, plasmacytoid monocytes, and the absence of neutrophils (13). With the use of histochemical stains and microbiologic cultures, the diagnosis of tuberculous lymphadenitis should be ruled out (3). Suppurative lymphadenitis can be differentiated by the presence of a large number of neutrophils which are not observed in Kikuchi’s isease. Frequently, Lupus lymphadenitis has "haematoxylin bodies" that have not been reported in patients with the Kikuchi disease (1). Histologically, abundant karyorrhectic debris and sheets of macrophages seen in the KFD have caused it to become highly similar to high- grade lymphoma. Therefore, distinguishing them is crucial due to the significant differences in their treatment and prognosis. The polymorphous inflammatory infiltrate and Reed-Sternberg cells in Hodgkin's lymphoma are highly characteristics and these can be immunophenotypically differentiated by CD15 staining (14). In nodal non-Hodgkin's lymphoma, the infarcted areas are generally rimmed by granulation tissue and may contain the ghosts of the malignant cells (1). The mainstay treatment for this disease is supportive care and symptomatic relief (2,3,11). Nonsteroidal Anti-inflammatory Drugs might be beneficial in painful lymph node enlargement and fever (13). Meanwhile, Corticosteroids are used in a more severe and generalized Kikuchi disease for cases with existing neurologic or hepatic involvement and cases with severe lupus-like syndrome (13). However, this patient was also observed to have hepatic involvement and a positive antinuclear antibody test. Corticosteroid may be used in less severe cases to help patients recover faster from their symptoms (10,11,13). In some cases of recurrent KFD and steroid-resistant patients, Hydroxychloroquine and intravenous immunoglobulin have successful been used in treating the patients (15). 

## Conclusion

Even though prognosis of the Kikuchi disease is good, these patients should remain under regular clinical surveillance due to subsequent development of systemic lupus erythematosus. Meanwhile, timely diagnosis of the Kikuchi disease is crucial to avoid extensive diagnostic procedures and inappropriate treatment modalities.
